# The amniotic fluid cell-free transcriptome in spontaneous preterm labor

**DOI:** 10.1038/s41598-021-92439-x

**Published:** 2021-06-29

**Authors:** Gaurav Bhatti, Roberto Romero, Nardhy Gomez-Lopez, Roger Pique-Regi, Percy Pacora, Eunjung Jung, Lami Yeo, Chaur-Dong Hsu, Mahendra Kavdia, Adi L. Tarca

**Affiliations:** 1 Perinatology Research Branch, Eunice Kennedy Shriver National Institute of Child Health and Human Development, National Institutes of Health, U.S. Department of Health and Human Services, Detroit, MI USA; 2 Department of Obstetrics and Gynecology, Wayne State University School of Medicine, Detroit, MI USA; 3 Department of Obstetrics and Gynecology, University of Michigan Health System, Ann Arbor, MI USA; 4 Department of Epidemiology and Biostatistics, College of Human Medicine, Michigan State University, East Lansing, MI USA; 5 Center for Molecular Medicine and Genetics, Wayne State University, Detroit, MI USA; 6 Detroit Medical Center, Detroit, MI USA; 7 Department of Obstetrics and Gynecology, Florida International University, Miami, FL USA; 8 Department of Biochemistry, Microbiology and Immunology, Wayne State University School of Medicine, Detroit, MI USA; 9 Department of Physiology, Wayne State University School of Medicine, Detroit, MI USA; 10 Department of Biomedical Engineering, Wayne State University College of Engineering, Detroit, MI USA

**Keywords:** Predictive markers, Microarrays, Transcriptomics, Pregnancy outcome, Prognostic markers, Preterm birth

## Abstract

The amniotic fluid (AF) cell-free RNA was shown to reflect physiological and pathological processes in pregnancy, but its value in the prediction of spontaneous preterm delivery is unknown. Herein we profiled cell-free RNA in AF samples collected from women who underwent transabdominal amniocentesis after an episode of spontaneous preterm labor and subsequently delivered within 24 h (n = 10) or later (n = 28) in gestation. Expression of known placental single-cell RNA-Seq signatures was quantified in AF cell-free RNA and compared between the groups. Random forest models were applied to predict time-to-delivery after amniocentesis. There were 2385 genes differentially expressed in AF samples of women who delivered within 24 h of amniocentesis compared to gestational age-matched samples from women who delivered after 24 h of amniocentesis. Genes with cell-free RNA changes were associated with immune and inflammatory processes related to the onset of labor, and the expression of placental single-cell RNA-Seq signatures of immune cells was increased with imminent delivery. AF transcriptomic prediction models captured these effects and predicted delivery within 24 h of amniocentesis (AUROC = 0.81). These results may inform the development of biomarkers for spontaneous preterm birth.

## Introduction

The World Health Organization defines preterm birth as the delivery of a neonate between 20 and 37 weeks of gestation^1^. Globally, 14.84 million preterm births and 1.1 million prematurity-related deaths occur each year^2,3^. The United States has seen the incidence of preterm birth continue to increase since 2014, and this rate has recently plateaued at nearly 10% since 2018^4^. Preterm birth is the leading cause of neonatal mortality (death within 28 days of delivery) and morbidity (e.g., neonatal respiratory morbidity and NICU triage/admission) as well as infant mortality (death before 5 years of age)^3,5^. Prematurely born infants are at an elevated risk of developing chronic diseases that may include neurological disorders (e.g., learning disabilities) and cardiovascular diseases later in life^6,7^. Preterm birth poses a substantial economic burden on society; approximately $26.2 billion are spent annually on the care of prematurely born infants^8^; hence, there is a sustained effort to develop strategies to prevent and reduce the impact of preterm birth. Although there has been success in reducing the mortality rate, other adverse neonatal outcomes associated with prematurity have remained consistently prevalent^9–11^.

Preterm birth is either iatrogenic (i.e., medically indicated in the event of disease, such as preeclampsia) or spontaneous following preterm labor or preterm pre-labor rupture of the membranes^12,13^. Spontaneous preterm labor accounts for most of the preterm births, characterized by multiple underlying etiologies culminating in the activation of a common pathway of labor that leads to spontaneous preterm delivery^13,14^. There is evidence to support a causal relationship between microbial-associated or sterile intra-amniotic inflammation and spontaneous preterm labor and birth^15–24^. Intra-amniotic inflammation, however, is responsible for only a subset of cases with spontaneous preterm labor and delivery, while most cases of preterm labor are considered to be idiopathic or have an unknown etiology.

To address the complex public health problem of preterm birth, the development of biomarkers for preterm labor is necessary^25–28^. Currently, risk modeling of preterm birth relies on maternal factors: a history of preterm birth^29^, of late miscarriage^30^ or cervical excisional surgery^31^, a sonographic short cervix^32^, a low customized cervical length percentile^33^ during the current pregnancy, amniotic fluid sludge^34^, and an abnormal cervical consistency index^35^. Biochemical markers, e.g., fFN^36^, PIGFBP-1^37^, PAMG-1^38^, inflammatory cytokines^39^, and cervical acetate^40^, as well as the vaginal microbiome^41^ and maternal blood transcriptome^42^, have also been suggested as predictive of preterm birth. Combinations of biomarkers have shown superior predictive performance compared to any single test^43^. However, given the syndromic nature of spontaneous preterm labor, there is a need to improve preterm birth prediction performance relative to current biomarkers^43,44^.

Amniotic fluid (AF) surrounds the developing fetus and is in continuous exchange with fetal organs and gestational tissues^45–47^; hence, this fluid is a rich source of potential biomarkers for preterm labor and birth^48^. The cell-free supernatant that remains upon removing the AF's cellular components reflects fetal well-being and pregnancy status^46,47,49–56^. Indeed, studies have described changes in the AF cell-free transcriptome in fetal genetic disorders, such as trisomy 18^50^, trisomy 21^49^, and Turner syndrome^52^, as well as in fetal growth restriction^54^ and preeclampsia^55^. Others have investigated the association between the AF cell-free transcriptome and maternal factors such as race, obesity, and smoking status^51,56^, as well as neonatal morbidity^53^; yet, the AF cell-free transcriptome in spontaneous preterm labor was not assessed in prior investigations.

To address the current knowledge gap, we performed whole transcriptome profiling of AF in mothers who underwent transabdominal amniocentesis after an episode of preterm labor and then utilized machine learning to predict the time-to-delivery after amniocentesis. Given the recent development of cell-type-specific signatures based on single-cell genomic studies of the placenta^57–61^ and the relevance of these signatures in identifying preeclampsia^58,62^ and preterm parturition^61^, we have also assessed the perturbations of these signatures in AF cell-free RNA.

## Results

### Demographic characteristics of the study participants

**Figure 1:**
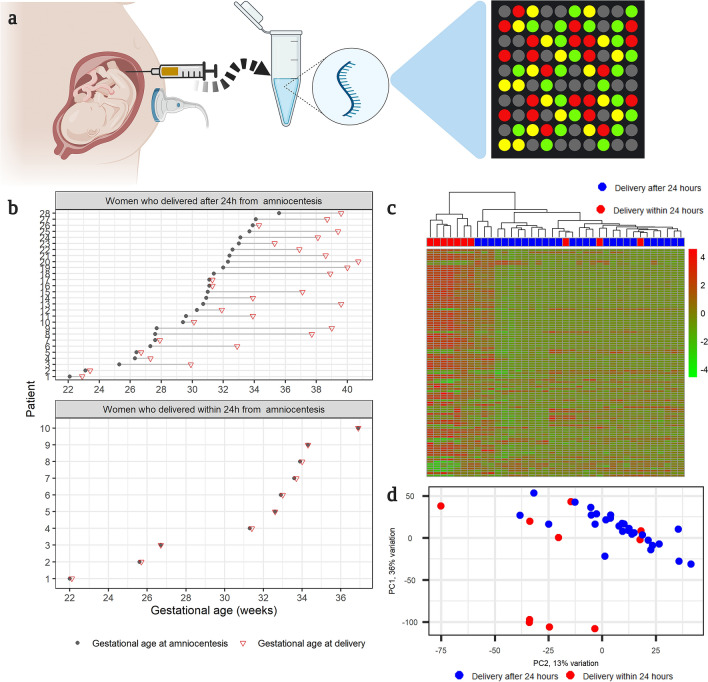
(**a**) Study Design (created with biorender.com). The cell-free transcriptome of amniotic fluid samples collected from women after an episode of preterm labor was quantified with microarrays. (**b**) Distribution of gestational age at sampling. Each line corresponds to a single mother, and each circle represents a sample. The green triangles mark the gestational age at delivery. (**c**) Unsupervised clustering. Heatmap shows the hierarchical clustering of samples based on the expression of the most variable genes. The R package, *pheatmap,* was used to generate the heatmap. (**d**) Principal component analysis of amniotic fluid cell-free RNA expression. All samples are depicted as their first and second principal components derived from the cell-free amniotic fluid transcriptome. The proportion of variance explained by each principal component is shown along the axis. The R/Bioconductor package, *PCAtools*, was used to calculate and plot the principal components.

We examined the AF cell-free transcriptome in samples collected from 38 women who had a transabdominal amniocentesis performed after an episode of preterm labor (Fig. 1a). Women were divided into two groups according to the interval from amniocentesis to delivery (Fig. 1b): (1) women who delivered within 24 h of amniocentesis (n = 10) and (2) women who delivered after 24 h from amniocentesis (n = 28). Table 1 presents a comparison of the clinical characteristics of women between the two groups.

**Table 1:** Demographic characteristics of the women included in the transcriptomics study.

	Time to delivery after amniocentesis ≤ 24 h (n = 10)	Time to delivery after amniocentesis > 24 h (n = 28)	p
Maternal age (years)	23 (22.2–31.8)	24.5 (22.8–28)	0.767
Body mass index (kg/m^2^)	24.8 (20.9–27.8)	22.4 (20.2–29.9)	0.792
Nulliparity	3/10 (30%)	4/28 (14.3%)	0.351
History of preterm birth	4/10 (40%)	10/28 (35.7%)	1
African American ethnicity	9/10 (90%)	24/28 (85.7%)	1
Gestational age at amniocentesis (weeks)	32.8 (27.8–33.8)	31 (27.6–32.4)	0.576
Amniotic fluid IL-6 ≥ 2.6 ng/ml	8/10 (80%)	8/28 (28.6%)	0.008
Positive amniotic fluid culture	2/9 (22.2%)	2/28 (7.1%)	0.244
Amniotic fluid WBC (cells/mm3) ≥ 50 cells/mm3	5/10 (50%)	0/28 (0%)	< 0.001
Amniotic fluid glucose (mg/dl) < 14 mg/dl	6/9 (66.7%)	1/28 (3.6%)	< 0.001
Gestational age at delivery (weeks)	32.8 (27.9–33.9)	34.8 (31–38.8)	0.092
Cesarean section	2/10 (20%)	3/28 (10.7%)	0.592
Birthweight (g)	1907.5 (1078.8–2037.5)	2142.5 (1587.5–2992.5)	0.047
Birthweight percentile	30.7 (18.5–50)	32.9 (21.7–48.3)	0.647
Antenatal corticosteroids	7/10 (70%)	26/28 (92.9%)	0.103
Tocolytic agents	4/10 (40%)	13/28 (46.4%)	1
Composite neonatal morbidity	7/10 (70%)	11/28 (39.3%)	0.144
Acute chorioamnionitis	5/9 (55.6%)	9/24 (37.5%)	0.442
Chronic chorioamnionitis	4/9 (44.4%)	7/24 (29.2%)	0.438

Continuous variables were compared with the Welch's t-test and are summarized as medians (interquartile range). Categorical variables are shown as number (%) and were compared by using a Fisher's exact test.

^a^Composite neonatal morbidity was defined as the presence of any of the following complications: 5 min Apgar score < 7, bronchopulmonary dysplasia, pulmonary hypoplasia, respiratory distress syndrome, necrotizing enterocolitis, intraventricular hemorrhage, periventricular leukomalacia, retinopathy of prematurity, neonatal sepsis, or NICU admission.

Women who delivered within 24 h of amniocentesis had smaller babies than those who delivered after 24 h (median birth weight: 1907.5 g vs. 2142.5 g, p = 0.047); yet, there were no differences in the birthweight percentiles. The AF interleukin (IL)-6 concentrations, frequency of AF glucose levels < 14 mg/dl, and frequency of AF white blood cell counts ≥ 50 cells/mm^3^ were higher in women who delivered within 24 h of amniocentesis than women who delivered after 24 h. Other fetal and maternal characteristics were similar between the two groups. Of importance, there was no significant difference in gestational age at amniocentesis between women who delivered within 24 h of amniocentesis compared to women who delivered after 24 h (median gestational age at amniocentesis: 32.8 weeks vs. 31 weeks, p > 0.5).

Amniotic fluid WBC count, IL-6 levels, and culture determinations are used in the clinical decisions in patients with preterm labor who undergo an amniocentesis. These decisions may include administering tocolytic agents to inhibit myometrial contractions, administering antenatal corticosteroids for fetal lung maturity, or early delivery in cases of severe infection/inflammation. However, none of the 38 women in this study had labor induced preterm, with only one case having labor induction at 40 weeks. Therefore, indicated early delivery was not a confounding factor in the analysis of cell-free RNA data. Clinical decision to administer tocolytic agents may affect the interval from amniocentesis to delivery, yet this was not the case here since tocolytic agent administration occurred at similar rates between the two groups (delivery ≤ 24 h from amniocentesis vs. > 24 h) (40% vs. 46.4%, respectively) (Table 1).

### Differential expression with imminent delivery after amniocentesis

Hierarchical clustering (Fig. 1c) based on the most variable genes and principal components analysis (Fig. 1d) shows an overall separation between the group of women who delivered within 24 h of amniocentesis and those who delivered after 24 h. The first principal component that captured 36% of the variation was correlated with gestational age at amniocentesis (Pearson correlation = 0.36, p = 0.027).

**Figure 2:**
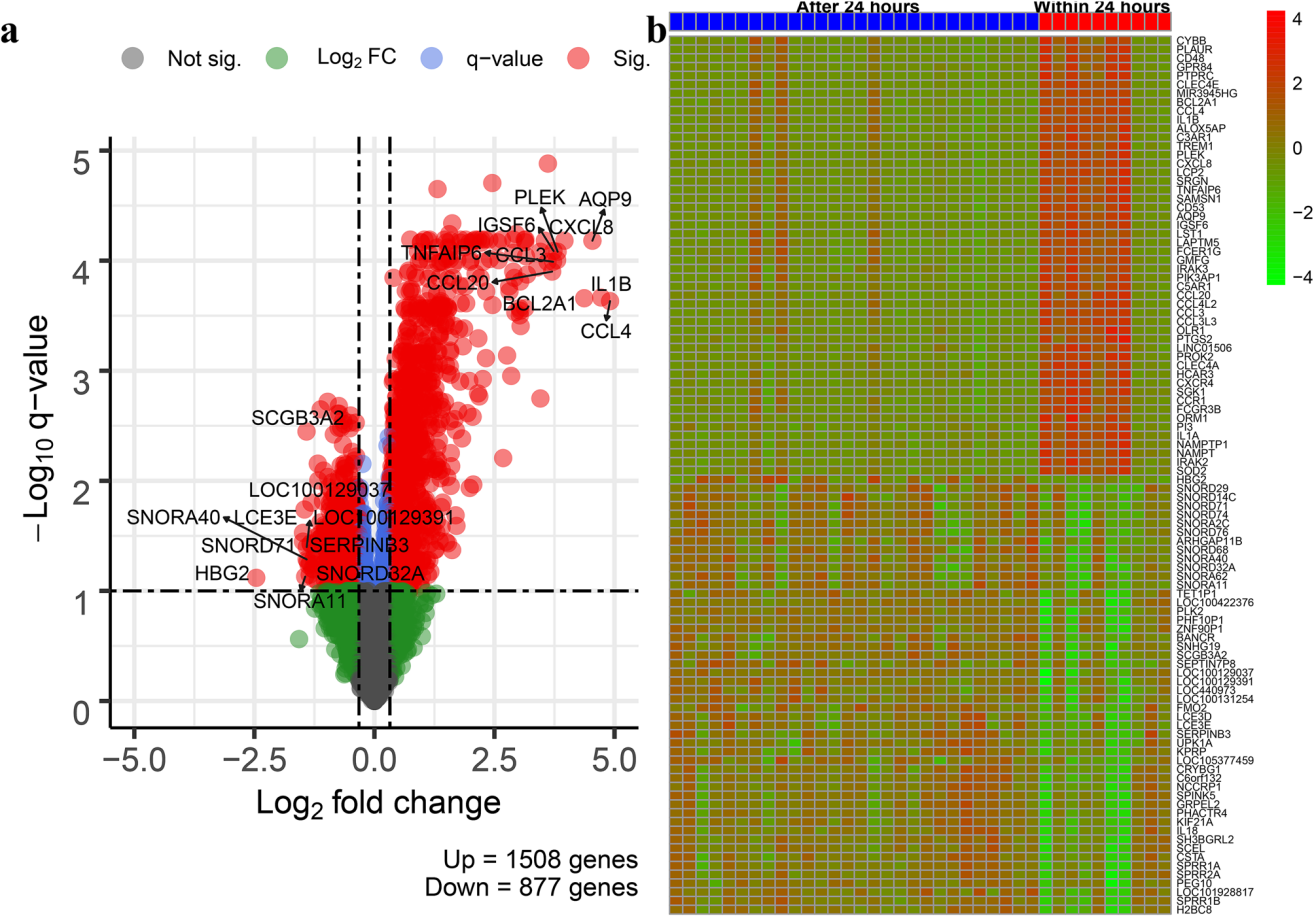
Differential expression analysis. The figure shows (**a**) the volcano plots of log_10_ transformed q- values against log_2_ transformed fold changes of all genes and (**b**) heatmaps based on the top 50 up-regulated and down-regulated genes for the comparison between women who delivered within 24 h of amniocentesis and those who delivered after 24 h. The R/Bioconductor packages, *EnhancedVolcano,* and *pheatmap*, were used to generate the volcano plot and heatmap, respectively.

The comparison of AF cell-free transcriptomes of women who delivered within 24 h of amniocentesis and those from women who delivered after 24 h showed differential expression (fold change > 1.25, q value < 0.1) of 2385 genes (1508 up-regulated and 877 down-regulated) (Table S1, Fig. 2).

The five most up-regulated genes were *CCL4* (C–C motif chemokine ligand 4), *IL1B* (interleukin 1 beta), *AQP9* (aquaporin 9), *BCL2A1* (BCL2 related protein A1), and *CXCL8* (C-X-C motif chemokine ligand 8). Functional analysis of up-regulated differentially expressed (DE) genes showed an over-representation of 1918 biological processes, 171 cellular components, and 143 molecular functions (Table S2). Most enriched biological processes were related to the inflammatory and immune responses to stimuli, e.g., pattern recognition receptor signaling pathway, leukocyte mediated immunity, NIK/NF-kappaB signaling, and cytokine-mediated signaling pathway. Significantly over-represented cellular components included terms related to extracellular region, membrane, cytoplasmic vesicle part, and I-kappaB/NF-kappaB complex. The over-represented molecular functions included receptor binding, receptor regulator activity, signaling receptor activity, and oxidoreductase activity, acting on NAD(P)H, oxygen as acceptor.

The most down-regulated genes with imminent delivery included *HBG2* (hemoglobin subunit gamma 2), *SNORD71* (small nucleolar RNA, C/D box 71), *LCE3E* (late cornified envelope 3E), *LOC100129037* (WEE1 homolog (S. pombe) pseudogene), and *SNORA11* (small nucleolar RNA, H/ACA box 11). Gene ontology enrichment analysis of down-regulated genes identified 492 biological processes, 151 cellular components, and 130 molecular functions (Table S3). The significant biological processes were related to nucleobase-containing compound metabolism, organelle organization, cell cycle, and cellular response to DNA damage. The most-enriched cellular components included nuclear part, organelle, catalytic complex, and cell junction. Protein binding, DNA binding, DNA-dependent ATPase activity, and N-acetyltransferase activity were among the most over-represented molecular functions among the down-regulated genes.

### Changes in cell-type and tissue-specific signatures in AF with imminent delivery

**Figure 3:**
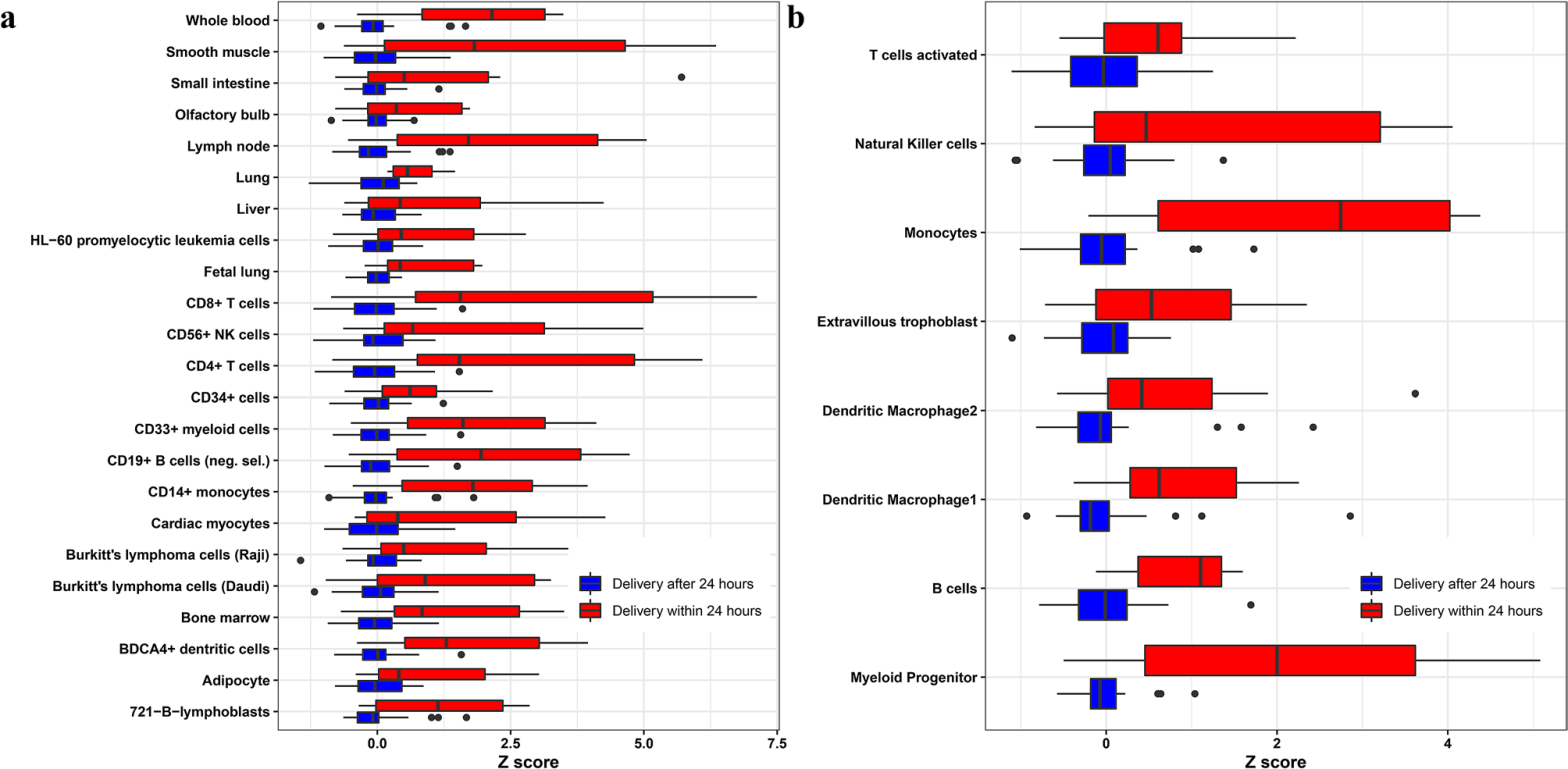
Expression of tissue-specific and placental single-cell RNA Seq signatures. For each (**a**) tissue and (**b**) placental single-cell signature, the expression of the top 20 most preferentially expressed genes was transformed into a Z score and averaged. The Z scores were compared between women who delivered within 24 h of amniocentesis and those who delivered after 24 h. Significant tissues and placental single-cell types (q value < 0.05) are shown in the figure.

To gain further insight into the meaning of the differential expression findings between the study groups, we compared the expression of gene sets defined based on their specificity to tissues and cell-types (according to the GNF Expression Atlas^63^). The average standardized expression of genes preferentially expressed in 23 tissues or cell-types was significantly increased in the AF cell-free transcriptome of women who delivered within 24 h of amniocentesis compared to women who delivered after 24 h (q < 0.05) (Fig. 3a). These included tissues and cell-type-specific signatures of organs (e.g., fetal lung, liver, and olfactory bulb) and immune system-related signatures (bone marrow, lymph nodes, whole blood, T cells, B cells, monocytes, and natural killer cells).

### Changes in placental scRNA-Seq signatures in AF with imminent delivery

By utilizing the same type of analysis described above for tissues, we have also analyzed changes in placental scRNA-Seq signatures derived from the placentas' three compartments (basal plate, placental villi, and chorio-amniotic membranes) in women with term labor or preterm labor^61^. There was a significant increase in the average expression of genes specific to monocytes, myeloid progenitor cells, dendritic macrophages, activated T cells, B cells, natural killer cells, and extravillous trophoblasts in women who delivered within 24 h from amniocentesis compared to those who delivered after 24 h (Fig. 3b).

### Prediction of time-to-delivery

**Figure 4:**
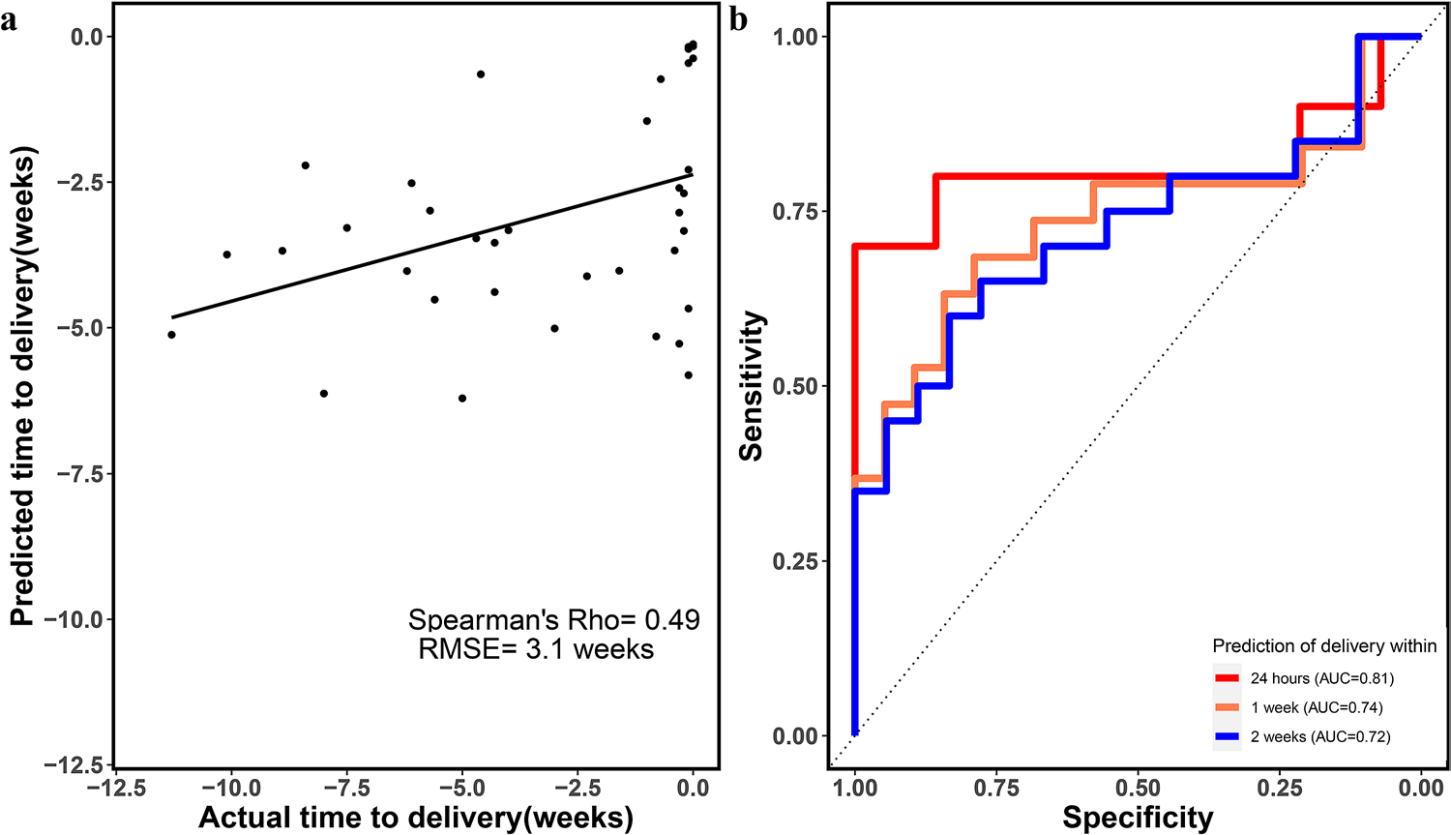
Prediction of time-to-delivery by cell-free amniotic fluid transcriptomics. (**a**) Cross-validation based predictions of time to delivery are plotted against actual values for all samples. (**b**) Receiver operating characteristic curve for the prediction of imminent delivery by estimates of the time-to-delivery (generated with the R package, *pROC*). *RMSE* root mean squared error.

Based on the differential expression analysis, we concluded that the AF cell-free transcriptome of women with preterm labor echoes the inflammatory response that precedes delivery. We hypothesized that predictive models that capture these effects should predict the time-to-delivery after amniocentesis. Our modeling strategy included the selection of RNAs that were most informative about the interval from amniocentesis to delivery in a multivariate evaluation, followed by random forest model fitting of gene expression data to predict time-to-delivery as a continuous variable. The cross-validated prediction of time-to-delivery by a transcriptomic model was significant, with a Spearman's correlation coefficient of 0.49 (p < 0.001) and a root-mean-square error (RMSE) of 3.1 weeks (Fig. 4a). When assessed as a binary outcome, prediction of delivery within 24 h of the procedure by the transcriptomic model time-to-delivery estimates was also significant as indicated by a receiver operating characteristic (ROC) curve analysis (Fig. 4b). The areas under the ROC curve (AUROC) for prediction of delivery within 24 h, 1 week, and 2 weeks were 0.81, 0.74, and 0.72, respectively.

**Figure 5:**
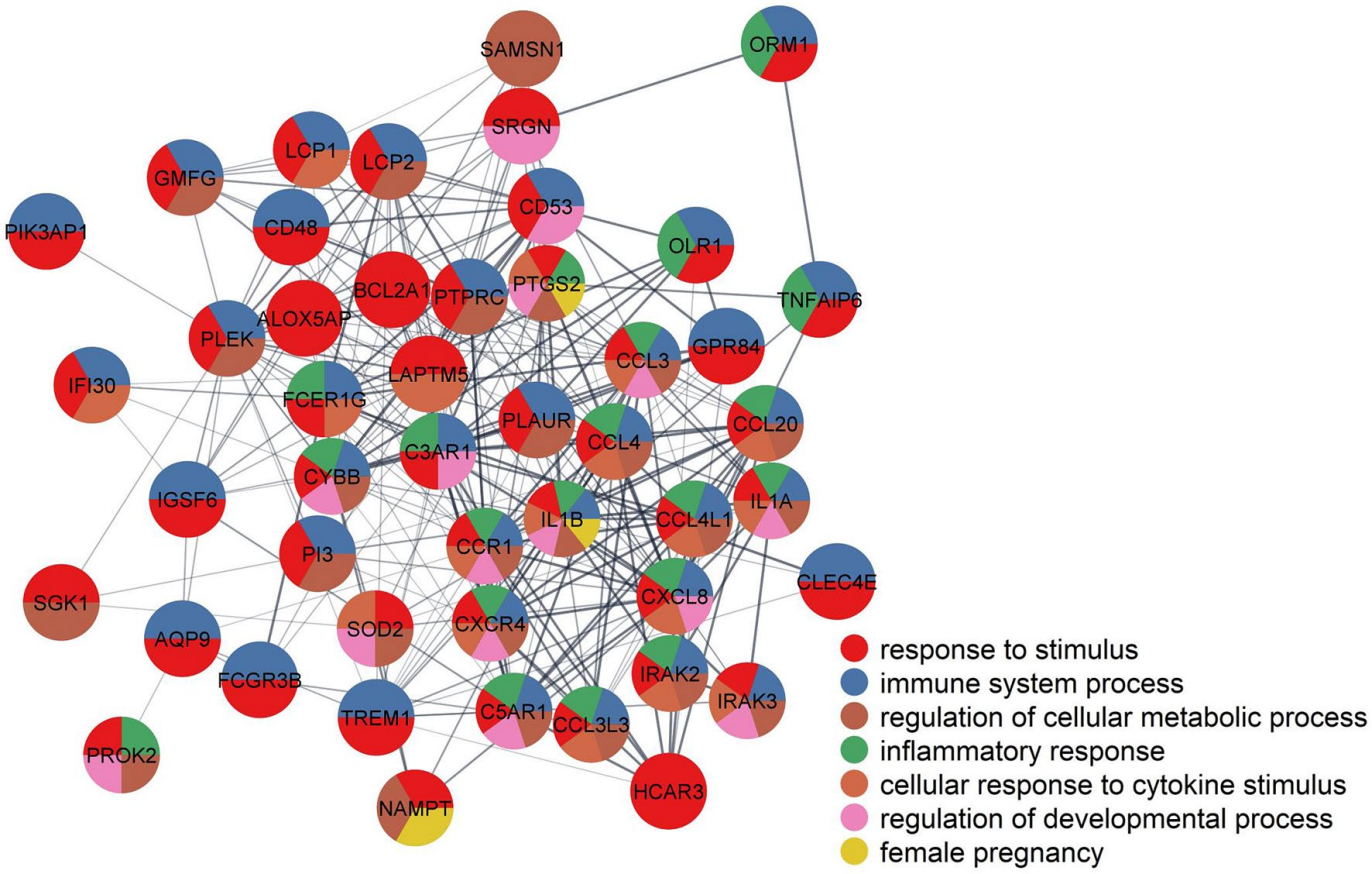
Protein–protein interaction network for the most predictive genes. Selected biological processes over-represented among the most predictive genes are shown in the pie charts. The network was created with stringApp v1.5.0 in Cytoscape v3.7.2.

To assess the robustness and reproducibility of genes selected as predictors during the cross-validation analysis, we calculated the average Jaccard similarity (0.82) and average kappa coefficient (0.9) between all sets of predictor genes identified across leave-one-out iterations. Based on Kursa's^64^ definition of significantly self-consistent selection, 53 of the most-predictive genes of time-to-delivery in AF samples are highlighted in Table 2. All 53 genes selected as predictors were up-regulated in the AF cell-free RNA of women who delivered within 24 h of amniocentesis compared to those who delivered later. Of note, 23 genes were selected in all iterations of leave-one-out cross-validation; these included *IL1B* (interleukin 1 beta), *CXCL8* (C-X-C motif chemokine ligand 8), *AQP9* (aquaporin 9), *PLEK* (pleckstrin), *BCL2A1* (BCL2 related protein A1), *CCL3* (C–C motif chemokine ligand 3), *CCL4* (C–C motif chemokine ligand 4), and *CCL20* (C–C motif chemokine ligand 20). Figure 5 shows a highly connected protein–protein interaction network built based on the corresponding 53 predictor genes. In this figure, several enriched Gene Ontology biological processes related to the immune and inflammatory responses are highlighted (q value < 0.05).

**Table 2:** Amniotic fluid cell free RNAs most predictive of time from amniocentesis to delivery.

Entrez	Symbol	Genename
10261	*IGSF6*	Immunoglobulin superfamily member 6
1230	*CCR1*	C–C motif chemokine receptor 1
2207	*FCER1G*	Fc fragment of IgE receptor Ig
241	*ALOX5AP*	Arachidonate 5-lipoxygenase activating protein
26253	*CLEC4E*	C-type lectin domain family 4 member E
3553	*IL1B*	Interleukin 1 beta
3576	*CXCL8*	C-X-C motif chemokine ligand 8
366	*AQP9*	Aquaporin 9
3937	*LCP2*	Lymphocyte cytosolic protein 2
5266	*PI3*	Peptidase inhibitor 3
5329	*PLAUR*	Plasminogen activator, urokinase receptor
5341	*PLEK*	Pleckstrin
597	*BCL2A1*	BCL2 related protein A1
6348	*CCL3*	C–C motif chemokine ligand 3
6351	*CCL4*	C–C motif chemokine ligand 4
6364	*CCL20*	C–C motif chemokine ligand 20
64092	*SAMSN1*	SAM domain, SH3 domain and nuclear localization signals 1
7130	*TNFAIP6*	TNF alpha induced protein 6
7805	*LAPTM5*	Lysosomal protein transmembrane 5
8843	*HCAR3*	Hydroxycarboxylic acid receptor 3
9535	*GMFG*	Glia maturation factor gamma
9560	*CCL4L2*	C–C motif chemokine ligand 4 like 2
963	*CD53*	CD53 molecule
54210	*TREM1*	Triggering receptor expressed on myeloid cells 1
719	*C3AR1*	Complement C3a receptor 1
1536	*CYBB*	Cytochrome b-245 beta chain
2215	*FCGR3B*	Fc fragment of IgG receptor IIIb
3552	*IL1A*	Interleukin 1 alpha
4973	*OLR1*	Oxidized low density lipoprotein receptor 1
731424	*MIR3945HG*	MIR3945 host gene
53831	*GPR84*	G protein-coupled receptor 84
60675	*PROK2*	Prokineticin 2
962	*CD48*	CD48 molecule
5743	*PTGS2*	Prostaglandin-endoperoxide synthase 2
7852	*CXCR4*	C-X-C motif chemokine receptor 4
414062	*CCL3L3*	C–C motif chemokine ligand 3 like 3
646309	*NAMPTP1*	Nicotinamide phosphoribosyltransferase pseudogene 1
6648	*SOD2*	Superoxide dismutase 2
5552	*SRGN*	Serglycin
654816	*NCF1B*	Neutrophil cytosolic factor 1B pseudogene
118788	*PIK3AP1*	Phosphoinositide-3-kinase adaptor protein 1
3656	*IRAK2*	Interleukin 1 receptor associated kinase 2
3936	*LCP1*	Lymphocyte cytosolic protein 1
5004	*ORM1*	Orosomucoid 1
7940	*LST1*	Leukocyte specific transcript 1
11213	*IRAK3*	Interleukin 1 receptor associated kinase 3
6446	*SGK1*	Serum/glucocorticoid regulated kinase 1
645965	*LOC645965*	Adipose differentiation-related protein pseudogene
728	*C5AR1*	Complement C5a receptor 1
10135	*NAMPT*	Nicotinamide phosphoribosyltransferase
10437	*IFI30*	IFI30 lysosomal thiol reductase
5788	*PTPRC*	Protein tyrosine phosphatase receptor type C
654817	*NCF1C*	Neutrophil cytosolic factor 1C pseudogene

The table consists of the gene symbol, gene name, and ENTREZ database identifier.

## Discussion

Spontaneous preterm labor is a syndrome with many etiologies and may involve intra-amniotic inflammation with or without microbial infection, oxidative stress, and placental dysfunction^14^. Accurate prediction and mitigation of spontaneous preterm birth are still challenging. Identification of symptomatic patients at the greatest risk of impending delivery allows obstetricians to implement prophylactic interventions and timely transfer into tertiary care centers and to guide antenatal therapy and postnatal care intended to reduce the risk of adverse outcomes the neonate^65–68^. Reassuring mothers that they are not at risk of imminent delivery also alleviates stress, thereby improving the likelihood of an uncomplicated pregnancy^69^.

We hypothesized that the proximity of amniotic fluid to the fetus and gestational tissues makes it an ideal source to explore the transcriptomic perturbations preceding delivery. Herein we have characterized for the first time AF cell-free transcriptomic changes and identified placental single-cell RNA signatures in women who present with an episode of preterm labor. These data could inform the development of biomarkers in subsequent studies based on minimally invasive samples, such as maternal blood.

The current study included women (n = 38) who had transabdominal amniocentesis performed after an episode of preterm labor, with some delivering on the same day (within 24 h, n = 10) and others delivering later in gestation (n = 28). All women who delivered within 24 h of amniocentesis had a preterm birth, whereas 57% (16/28) of women delivering after 24 h had a preterm birth. Intra-amniotic inflammation (IL-6 ≥ 2600 pg/ml) was diagnosed in 80% (8/10) of women who delivered within 24 h of amniocentesis and in 29% (8/28) of women who gave birth later in gestation. Consistent with this observation, comparison of AF cell-free transcriptomes between the two groups revealed an up-regulation of genes involved in the immune system's innate and adaptive components, including myeloid leukocyte activation, regulation of complement activation, toll-like receptor signaling pathway, natural killer cell-mediated cytotoxicity, B-cell-mediated immunity, and T-cell-mediated cytotoxicity. The expression of gene signatures defining cell-types (myeloid cells, monocytes, T cells, B cells, and natural killer cells) and tissues involved in the immune response also increased in the amniotic fluid of women with imminent preterm delivery. Previous studies have shown that the fetal or maternal origin of the amniotic fluid immune response depends on the gestational age, with fetal origin suggested during early preterm gestation and maternal origin later in pregnancy^70–73^.

Genes having a lower expression close to delivery were enriched for intra-cellular biological processes, e.g., cellular macromolecule metabolic process, organelle organization, cytoskeleton organization, cell cycle, and embryonic development. Thus, we observed a shift in the amniotic fluid milieu from one that reflects fetal organ maturation and growth to a pro-inflammatory phenotype induced by the stimulation of a maternal and or a fetal immune response as delivery approached. The activation of the immune response was accompanied by the increased expression of genes coding for pro-inflammatory cytokines (e.g. *IL-1α*, *IL-1β*, and *IL-6*), chemokines (e.g. *CCL20*, *CXCL5*, *CCL5*, and *CXCL8*), matrix metalloproteases (e.g. *MMP1*, *MMP8*, and *MMP9*), nuclear factor kappa B (*NFKB1* and *NFKB2*), and prostaglandin-endoperoxide synthase 2, all of which have been implicated in the pathogenesis of the preterm parturition syndrome^18,74^. Taken together, these results suggest that the activation of fetal and maternal immune responses acts as the trigger for preterm parturition in women who delivered within 24 h of amniocentesis. This response may be triggered when microorganisms invade the amniotic cavity (intra-amniotic infection/inflammation) or even in the absence of detectable microbes (sterile intra-amniotic inflammation)^19^. We did not apply the molecular microbiological techniques that reliably discriminate between these two conditions^75^. However, a previous report suggests that sterile intra-amniotic inflammation is more prevalent than intra-amniotic infection/inflammation in preterm deliveries^19^. Sterile intra-amniotic inflammation is thought to be initiated by danger signals, or alarmins, derived from cellular stress or necrotic release of intracellular matter into the intra-amniotic space^20,76–80^. This process involves the activation of the NLRP3 inflammasome^22,23,81–86^. Indeed, we observed an increased expression of the putative activators, components, and effectors of the inflammasome, such as *IL-1α*, baculoviral IAP repeat containing 3, guanylate binding protein 5, *NLRP3*, caspase-1, and IL-1β^84,87,88^.

In the current study, we also interrogated the second-trimester AF cell-free transcriptome to identify predictors of time-to-delivery after amniocentesis and to evaluate their predictive performance in risk stratification of women who present with symptoms of preterm labor. Previously, Ngo et al.^42^ developed a transcriptomic model of time-to-delivery based on longitudinal maternal blood cell-free RNA, and reported that a term-delivery-based model does not predict gestational age at delivery in women with preterm birth (RMSE, 11.4 weeks). Herein, we used the AF cell-free transcriptomic data from women with preterm labor to train robust machine-learning models to estimate the interval from amniocentesis to delivery. The cross-validated transcriptomic models showed a significant prediction (Spearman’s correlation 0.5, RMSE 3.1 weeks). When the continuous time-to-delivery estimates from the transcriptomic model were translated into binary predictions of delivery within 24 h, 1 week, and 2 weeks, the risk of imminent delivery was predicted with an AUROC of 0.81, 0.74, and 0.72, respectively. These results point to the AF cell-free transcriptome's potential to predict pregnancy duration after preterm labor.

A parsimonious set of 53 genes was reliably retained as predictors during cross-validation. These genes were up-regulated at delivery, and functional analysis identified biological processes related to an immune/inflammatory response to a stimulus. Biomarkers targeting these processes were identified in predictive models based on a whole-blood transcriptome^42,89,90^. The pro-inflammatory cytokines, *IL-1β*, *CXCL8*, *CCL3*, *CCL4*, and *CCL20*, were among the strongest predictors selected in all iterations of leave-one-out-cross-validation. Several studies have shown an increase in amniotic fluid concentrations of proteins encoded by these genes in women with preterm labor due to intra‐amniotic inflammation/infection^20,91–94^. A causal role in preterm birth has been established for IL-1β in animal models^15,95^. We have reported on the ability of these cytokine concentrations in the amniotic fluid in predicting the risk of early preterm delivery^94^. Other strong predictors included pleckstrin (*PLEK*), B-cell lymphoma 2-related protein A1 (*BCL2A1*), Solute Carrier Family 34 Member 2 (*SLC34A2*), Aquaporin 9 (*AQP9*), and *TNFAIP6*. Upon phosphorylation by protein kinase C, PLEK increases cytokine secretion in phagocytes and contributes as an adaptor to the microbicidal activity in neutrophils^96,97^. BCL2A1 is an anti-apoptotic protein shown to prolong chorio-decidual neutrophil survival in preterm rhesus macaques in an IL-1-dependent manner^98^. *SLC34A2* gene is expressed in alveolar type II cells of the fetal lung and may be involved in the cellular uptake of phosphate to produce surfactants^99^. However, the expression of genes coding for surfactant proteins was not significantly different between women delivering within 24 h of amniocentesis and women who delivered after 24 h, indicating that fetal lung maturity may not be related to imminent delivery in this study. Instead, the overexpression of *SLC34A2*, as well as *AQP9*, may reflect the metabolic adaptations needed to sustain the immune response^100^.

Of note, although the current study does not have immediate and direct consequences on the management of spontaneous preterm labor, it provides an RNA-level signature that could be correlated in the future with maternal blood omics data, allowing for the development of non-invasive approaches to risk assessment. Similar to the use of amniotic fluid IL6^101–103^, patients considered at high risk of imminent premature delivery could benefit from currently available interventions. These include the administration of antenatal corticosteroids to accelerate fetal lung maturity^66,104^ and tocolytics treatment to inhibit myometrium contractions, and eventually allow in utero transfer to tertiary neonatal intensive care units to provide better care to prematurely born neonate^67,68,104,105^.

### Strengths and limitations

This study is the first to describe the cell-free transcriptome of AF in women with spontaneous preterm labor. The main limitation of this study is that no additional targeted validation studies were performed. However, our previously reported in-silico analysis^56^ based on the same sample collection, RNA extraction, and expression quantification in the same population demonstrated that gestational age-specific effects in the transcriptome strongly correlate with independent reports based on samples from women of different ethnic backgrounds who were not in labor than those studied herein, for which most women self-identified as African-American. The moderate sample size and cross-validation strategy enabled the robust evaluation of predictive analytic approaches and the identification of a parsimonious set of candidate cell-free mRNAs.

## Conclusion

The changes in the AF cell-free transcriptome in women who delivered within 24 h of amniocentesis compared to those who delivered later in gestation indicated the establishment of an inflammatory milieu in the intra-amniotic space in response to a pathologic stimulus. Placental single-cell-specific gene signatures of critical immune cell types were also dysregulated in these women. These effects are critical components captured by the transcriptomic models predicting the duration of gestation after transabdominal amniocentesis.

## Materials and methods

### Study design

Pregnant women were enrolled into a prospective longitudinal study at the Center for Advanced Obstetrical Care and Research of the Perinatology Research Branch, *Eunice Kennedy Shriver* National Institute of Child Health and Human Development (NICHD), National Institutes of Health, U.S. Department of Health and Human Services, in the Detroit Medical Center and Wayne State University. We designed a retrospective cross-sectional study from this cohort to include women who underwent transabdominal amniocentesis after an episode of preterm labor. We excluded cases with multiple gestations and genetic anomalies. The final dataset included 38 AF samples from 12 women who went on to deliver at term and 26 women who delivered preterm. Of the 38 women, 37 delivered after the spontaneous onset of labor, with augmentation of labor required in 7 women. Labor was only induced in one term pregnancy (40.7 weeks) more than 8 weeks after amniocentesis. There were no cases of preterm prelabor rupture of the membranes. All women provided written consent for the use of biological specimens and metadata in research prior to sample collection. The Institutional Review Boards of Wayne State University and NICHD approved the study protocol.

### Clinical definitions

Gestational age was determined based on the date of the last menstrual period and a first or early second-trimester ultrasound examination. Term labor was defined as the presence of regular uterine contractions with a frequency of at least one every 10 min and cervical changes occurring after 37 weeks of gestation^106^. Spontaneous preterm labor was defined as the spontaneous onset of labor with intact membranes before 37 weeks of gestation.

### Amniotic fluid samples

Obstetricians used a 22-gauge needle to withdraw AF transabdominally while monitoring with ultrasound under antiseptic conditions. Amniotic fluid was immediately transported to the research laboratory in a capped sterile syringe. Amniotic fluid was centrifuged at 350×*g* and the supernant (5 ml) was immediately stored at – 80 °C^46^.

### RNA extraction

RNA was extracted from the AF samples with the Plasma/Serum RNA Purification Maxi Kit (Cat. No. 56200; Norgen Biotek, Thorold, ON, Canada) by following the manufacturer's protocol that included the optional DNase treatment. We applied the RNA Clean & Concentrator-5 Kit (Cat. No. R1015; Zymo Research, Irvine, CA, USA) to improve RNA quality and to concentrate each sample in a volume of 12 μl. RNA concentration and quality were assessed by using a DropSense spectrophotometer (Trinean NV, Gent, Belgium) and the Agilent 2200 Tapestation (Agilent Technologies, Santa Clara, CA, USA), respectively.

### Transcriptome profiling

Following the manufacturer’s protocol, we reverse-transcribed and amplified 10 ng of RNA to cDNA by using the GeneChip WT Pico Reagent Kit (Affymetrix, Santa Clara, CA, USA). Using the same kit, fragmentation and labeling of 5.5 μg sense-stranded cDNA were performed. Hybridization of 200 μl of the hybridization cocktail was performed onto the GeneChip Human Transcriptome Array 2.0 (Affymetrix, Santa Clara, CA, USA) at 45 °C, 60 rpm for 16 h in a hybridization oven. Washing and staining of the arrays were done on a GeneChip Fluidics Station 450 (Affymetrix, Santa Clara, CA, USA) and scanning was performed with a GeneChip Scanner 3000 (Affymetrix, Santa Clara, CA, USA). GeneChip Command Console Software (Affymetrix, Santa Clara, CA, USA) was used to generate the raw intensities from the array images. The University of Michigan Advanced Genomics Core conducted microarray profiling.

## Data analysis

### Clinical characteristics

Continuous and categorical variables were compared between groups by using the Welch's t test^107^ and the Fisher's exact test^108^, respectively. A nominal p value < 0.05 was considered statistically significant.

### Microarray data preprocessing

The Robust Multi-array Average^109^ method implemented in the R/Bioconductor's *oligo* package^110^ was applied to background correct, normalize, and summarize the raw probe-level microarray data into gene-level expression summaries based on probe-to-gene assignment provided by a custom chip definition file^111,112^. Since samples were profiled in multiple batches, we used the *removeBatchEffect* function of R/Bioconductor's *limma* package^113^ to correct batch effects. To assess sources of variability in the transcriptomic data, we conducted principal component analysis on the complete set of 32,907 genes.

### Differential expression analysis

We compared the AF cell-free transcriptome between groups by fitting linear models implemented in the *limma*^113^ package. A minimum fold change of 1.25-fold and a false discovery rate adjusted p value (q value) < 0.1 determined the statistical significance. We summarized the results of differential expression analysis with volcano plots and heatmaps. A hypergeometric test implemented in the *GOstats* package^114^ was used to identify significantly enriched Gene Ontology^115^ biological processes, molecular functions, and cellular components among the differentially expressed genes (q-value < 0.05).

### Tissue-specific and single-cell-specific expressions

Tissue-specific genes were defined as those having a median expression 30 times higher in a given tissue than all other tissues in the GNF Gene Expression Atlas^63^. Genes specific to a placental cell type were defined based on the single-cell RNA-Seq analyses^61^.

The log_2_ transformed expression values for each gene were standardized by subtracting the mean and dividing by the standard deviation calculated from the reference study group (term delivery)^56^. The standardized values referred to as Z scores were then averaged over the top 20 genes specific to a tissue or a single-cell type. We compared the average Z scores between groups by fitting a linear model. A q value of less than 0.05 was considered significant.

### Random forest prediction models

Random forest is an ensemble-supervised, machine-learning algorithm for classification and regression tasks^116^ suitable for high-dimensional data^117,118^. Unlike other methods, random forests are robust enough to feature transformation and parameter tuning while being computationally efficient^119^. Random forests consistently rank among the top performers in studies evaluating and comparing different supervised machine-learning algorithms^120^. They provide an unbiased measure of the predictor variable importance based on out-of-bag samples (observations not included in fitting individual decision trees)^116^. The R package, *randomForest*^121^*,*was used to fit random forest models with 1000 trees.

### Feature selection for the random forest models

Before fitting the random forest models, a preliminary multi-variable feature selection procedure was applied based on the sparse Hilbert–Schmidt independence criterion (SHS)^122^. SHS relies on the kernel-based Hilbert–Schmidt independence criterion (HSIC) to measure the relationship between the gene expression data and the response. By introducing penalties for the number of selected features, SHS chooses a parsimonious set of genes that maximizes the dependence with the response variable while taking into account the correlation between genes. We used the algorithm implementation provided by the authors of SHS to perform this analysis, using MATLAB (version R2018a).

### Prediction performance assessment

We used leave-one-out cross-validation to evaluate the predictive performance of the transcriptomic models of time from amniocentesis to delivery. At each iteration in the cross-validation, both feature selection and random forest model fitting were performed. Prediction performance metrics included Spearman’s correlation coefficient and root-mean-square error (RMSE). We also calculated the AUROC curves by using the predicted time-to-delivery as a surrogate of the inverse of the risk of delivery within 24 h, 1 week, and 2 weeks after amniocentesis.

To retain a final set of the most informative genes for the prediction of time-to-delivery, we employed the strategy of Kursa 2014^64^ based on the frequency of gene selection across different partitions of the data. Significantly enriched Gene Ontology biological processes were identified among the most predictive genes by using the *GOstats*^114^ package.

### The network of protein–protein interactions

To gain insight into the relations among genes predictive of time-to-delivery in AF samples, we constructed a protein–protein interaction network representation of these genes by using the stringApp 1.5.0^123^ in Cytoscape 3.7.2^124^. An edge between two nodes (genes) was defined based on a protein–protein interaction confidence score > 0.4. The network's most inter-connected subnetwork was retained, and genes were annotated to significantly enriched biological processes.

## Supplementary Information

Supplementary Information 1.

Supplementary Information 2.

Supplementary Information 3.

## References

[1] WHO. *ICD-11 for Mortality and Morbidity Statistics*. https://icd.who.int/browse11/l-m/en#/http://id.who.int/icd/entity/1726201225 (2018).

[2] Blencowe H (2012). National, regional, and worldwide estimates of preterm birth rates in the year 2010 with time trends since 1990 for selected countries: A systematic analysis and implications. Lancet.

[3] Liu L (2016). Global, regional, and national causes of under-5 mortality in 2000–15: An updated systematic analysis with implications for the sustainable development goals. Lancet.

[4] Martin JA, Hamilton BE, Osterman MJK, Driscoll AK (2019). Births: Final Data for 2018.

[5] Brown HK, Speechley KN, Macnab J, Natale R, Campbell MK (2014). Neonatal morbidity associated with late preterm and early term birth: The roles of gestational age and biological determinants of preterm birth. Int. J. Epidemiol..

[6] Mwaniki MK, Atieno M, Lawn JE, Newton CR (2012). Long-term neurodevelopmental outcomes after intrauterine and neonatal insults: A systematic review. Lancet.

[7] Raju TNK (2017). Long-term healthcare outcomes of preterm birth: An executive summary of a conference sponsored by the National Institutes of Health. J. Pediatr..

[8] Behrman RE, Butler AS (2007). Preterm birth: causes, consequences, and prevention.

[9] Horbar JD (2012). Mortality and neonatal morbidity among infants 501 to 1500 grams from 2000 to 2009. Pediatrics.

[10] Grisaru-Granovsky S (2014). Population-based trends in mortality and neonatal morbidities among singleton, very preterm, very low birth weight infants over 16 years. Early Hum. Dev..

[11] Hug L, Alexander M, You D, Alkema L, Estimation, U. I.-a. G. f. C. M (2019). National, regional, and global levels and trends in neonatal mortality between 1990 and 2017, with scenario-based projections to 2030: A systematic analysis. Lancet Glob. Health.

[12] Moutquin JM (2003). Classification and heterogeneity of preterm birth. BJOG.

[13] Goldenberg RL, Culhane JF, Iams JD, Romero R (2008). Epidemiology and causes of preterm birth. Lancet.

[14] Romero R, Dey SK, Fisher SJ (2014). Preterm labor: One syndrome, many causes. Science.

[15] Romero R, Mazor M, Tartakovsky B (1991). Systemic administration of interleukin-1 induces preterm parturition in mice. Am. J. Obstet. Gynecol..

[16] Romero R, Tartakovsky B (1992). The natural interleukin-1 receptor antagonist prevents interleukin-1-induced preterm delivery in mice. Am. J. Obstet. Gynecol..

[17] Romero R (2001). The role of infection in preterm labour and delivery. Paediatr. Perinat. Epidemiol..

[18] Romero R (2006). The preterm parturition syndrome. BJOG.

[19] Romero R (2014). Prevalence and clinical significance of sterile intra-amniotic inflammation in patients with preterm labor and intact membranes. Am. J. Reprod. Immunol..

[20] Romero R (2015). Evidence of perturbations of the cytokine network in preterm labor. Am. J. Obstet. Gynecol..

[21] Gomez-Lopez N (2016). Intra-amniotic administration of HMGB1 induces spontaneous preterm labor and birth. Am. J. Reprod. Immunol..

[22] Gomez-Lopez N (2019). Inhibition of the NLRP3 inflammasome can prevent sterile intra-amniotic inflammation, preterm labor/birth, and adverse neonatal outcomes†. Biol. Reprod..

[23] Faro J (2019). Intra-amniotic inflammation induces preterm birth by activating the NLRP3 inflammasome†. Biol. Reprod..

[24] Motomura K (2020). The alarmin interleukin-1alpha causes preterm birth through the NLRP3 inflammasome. Mol. Hum. Reprod..

[25] Green NS (2005). Research agenda for preterm birth: Recommendations from the March of Dimes. Am. J. Obstet. Gynecol..

[26] Gracie S (2011). An integrated systems biology approach to the study of preterm birth using "-omic" technology—a guideline for research. BMC Pregnancy Childbirth.

[27] Althabe F, Howson CP, Kinney M, Lawn J, World Health Organization (2012). 1 Online Resource (2 PDF files (xi, 112 Pages)).

[28] NICHD. *Preterm Labor and Birth: Research Activities and Scientific Advances*. https://www.nichd.nih.gov/health/topics/preterm/researchinfo/activities (2018).

[29] Laughon SK, Albert PS, Leishear K, Mendola P (2014). The NICHD consecutive pregnancies study: Recurrent preterm delivery by subtype. Am. J. Obstet. Gynecol..

[30] Oliver-Williams C, Fleming M, Wood AM, Smith G (2015). Previous miscarriage and the subsequent risk of preterm birth in Scotland, 1980–2008: A historical cohort study. BJOG.

[31] Sadler L, Saftlas A (2007). Cervical surgery and preterm birth. J. Perinat. Med..

[32] Souka AP (2011). Cervical length changes from the first to second trimester of pregnancy, and prediction of preterm birth by first-trimester sonographic cervical measurement. J. Ultrasound Med..

[33] Gudicha DW (2020). Personalized assessment of cervical length improves prediction of spontaneous preterm birth: A standard and a percentile calculator. Am. J. Obstet. Gynecol..

[34] Kusanovic JP (2007). Clinical significance of the presence of amniotic fluid 'sludge' in asymptomatic patients at high risk for spontaneous preterm delivery. Ultrasound Obstet. Gynecol..

[35] Baños N (2018). Mid-trimester sonographic cervical consistency index to predict spontaneous preterm birth in a low-risk population. Ultrasound Obstet. Gynecol..

[36] Hezelgrave NL, Shennan AH (2016). Quantitative fetal fibronectin to predict spontaneous preterm birth: A review. Womens Health (Lond.).

[37] Conde-Agudelo A, Romero R (2016). Cervical phosphorylated insulin-like growth factor binding protein-1 test for the prediction of preterm birth: A systematic review and metaanalysis. Am. J. Obstet. Gynecol..

[38] Melchor JC (2018). Predictive performance of PAMG-1 vs fFN test for risk of spontaneous preterm birth in symptomatic women attending an emergency obstetric unit: Retrospective cohort study. Ultrasound Obstet. Gynecol..

[39] Wei SQ, Fraser W, Luo ZC (2010). Inflammatory cytokines and spontaneous preterm birth in asymptomatic women: A systematic review. Obstet. Gynecol..

[40] Amabebe E (2016). Cervicovaginal fluid acetate: A metabolite marker of preterm birth in symptomatic pregnant women. Front. Med. (Lausanne).

[41] Fettweis JM (2019). The vaginal microbiome and preterm birth. Nat. Med..

[42] Ngo TTM (2018). Noninvasive blood tests for fetal development predict gestational age and preterm delivery. Science.

[43] Suff N, Story L, Shennan A (2019). The prediction of preterm delivery: What is new?. Semin. Fetal Neonatal Med..

[44] Oskovi Kaplan ZA, Ozgu-Erdinc AS (2018). Prediction of preterm birth: Maternal characteristics, ultrasound markers, and biomarkers: An updated overview. J. Pregnancy.

[45] Underwood MA, Gilbert WM, Sherman MP (2005). Amniotic fluid: Not just fetal urine anymore. J. Perinatol..

[46] Larrabee PB (2005). Global gene expression analysis of the living human fetus using cell-free messenger RNA in amniotic fluid. JAMA.

[47] Zwemer LM, Bianchi DW (2015). The amniotic fluid transcriptome as a guide to understanding fetal disease. Cold Spring Harb. Perspect Med..

[48] Kamath-Rayne BD, Smith HC, Muglia LJ, Morrow AL (2014). Amniotic fluid: The use of high-dimensional biology to understand fetal well-being. Reprod. Sci..

[49] Slonim DK (2009). Functional genomic analysis of amniotic fluid cell-free mRNA suggests that oxidative stress is significant in Down syndrome fetuses. Proc. Natl. Acad. Sci. USA.

[50] Koide K (2011). Transcriptomic analysis of cell-free fetal RNA suggests a specific molecular phenotype in trisomy 18. Hum. Genet..

[51] Edlow AG (2014). Maternal obesity affects fetal neurodevelopmental and metabolic gene expression: A pilot study. PLoS One.

[52] Massingham LJ (2014). Amniotic fluid RNA gene expression profiling provides insights into the phenotype of Turner syndrome. Hum. Genet..

[53] Kamath-Rayne BD (2015). Systems biology evaluation of cell-free amniotic fluid transcriptome of term and preterm infants to detect fetal maturity. BMC Med. Genom..

[54] Cho HY (2018). Functional analysis of cell-free RNA using mid-trimester amniotic fluid supernatant in pregnancy with the fetal growth restriction. Medicine (Baltimore).

[55] Jung YW (2019). Global gene expression analysis of cell-free RNA in amniotic fluid from women destined to develop preeclampsia. Medicine (Baltimore).

[56] Tarca AL (2020). Amniotic fluid cell-free transcriptome: A glimpse into fetal development and placental cellular dynamics during normal pregnancy. BMC Med. Genom..

[57] Nelson AC, Mould AW, Bikoff EK, Robertson EJ (2016). Single-cell RNA-seq reveals cell type-specific transcriptional signatures at the maternal-foetal interface during pregnancy. Nat. Commun..

[58] Tsang JCH (2017). Integrative single-cell and cell-free plasma RNA transcriptomics elucidates placental cellular dynamics. Proc. Natl. Acad. Sci. USA.

[59] Pavličev M (2017). Single-cell transcriptomics of the human placenta: Inferring the cell communication network of the maternal-fetal interface. Genome Res..

[60] Vento-Tormo R (2018). Single-cell reconstruction of the early maternal-fetal interface in humans. Nature.

[61] Pique-Regi R (2019). Single cell transcriptional signatures of the human placenta in term and preterm parturition. Elife.

[62] Tarca AL (2020). Maternal whole blood mRNA signatures identify women at risk of early preeclampsia: A longitudinal study. J. Matern. Fetal Neonatal Med..

[63] Su AI (2004). A gene atlas of the mouse and human protein-encoding transcriptomes. Proc. Natl. Acad. Sci. USA.

[64] Kursa MB (2014). Robustness of random forest-based gene selection methods. BMC Bioinform..

[65] Newnham JP (2014). Strategies to prevent preterm birth. Front. Immunol..

[66] Travers CP (2017). Exposure to any antenatal corticosteroids and outcomes in preterm infants by gestational age: Prospective cohort study. BMJ.

[67] Aboudi D, Shah SI, La Gamma EF, Brumberg HL (2018). Impact of neonatologist availability on preterm survival without morbidities. J. Perinatol..

[68] Helenius K (2019). Association of early postnatal transfer and birth outside a tertiary hospital with mortality and severe brain injury in extremely preterm infants: Observational cohort study with propensity score matching. BMJ.

[69] Dunkel Schetter C, Tanner L (2012). Anxiety, depression and stress in pregnancy: Implications for mothers, children, research, and practice. Curr. Opin. Psychiatry.

[70] Gomez-Lopez N (2017). Are amniotic fluid neutrophils in women with intraamniotic infection and/or inflammation of fetal or maternal origin?. Am. J. Obstet. Gynecol..

[71] Gomez-Lopez N (2019). The origin of amniotic fluid monocytes/macrophages in women with intra-amniotic inflammation or infection. J. Perinat. Med..

[72] Gomez-Lopez N (2019). Fetal T cell activation in the amniotic cavity during preterm labor: A potential mechanism for a subset of idiopathic preterm birth. J. Immunol..

[73] Gomez-Lopez N (2020). RNA sequencing reveals diverse functions of amniotic fluid neutrophils and monocytes/macrophages in intra-amniotic infection. J. Innate Immun..

[74] Vrachnis N (2012). Review: Impact of mediators present in amniotic fluid on preterm labour. In Vivo.

[75] Romero R (2014). A novel molecular microbiologic technique for the rapid diagnosis of microbial invasion of the amniotic cavity and intra-amniotic infection in preterm labor with intact membranes. Am. J. Reprod. Immunol..

[76] Romero R (1992). Interleukin-1 alpha and interleukin-1 beta in preterm and term human parturition. Am. J. Reprod. Immunol..

[77] Romero R (2011). Damage-associated molecular patterns (DAMPs) in preterm labor with intact membranes and preterm PROM: A study of the alarmin HMGB1. J. Matern. Fetal Neonatal Med..

[78] Romero R (2012). Clinical chorioamnionitis is characterized by changes in the expression of the alarmin HMGB1 and one of its receptors, sRAGE. J. Matern Fetal Neonatal Med..

[79] Friel LA (2007). The calcium binding protein, S100B, is increased in the amniotic fluid of women with intra-amniotic infection/inflammation and preterm labor with intact or ruptured membranes. J. Perinat. Med..

[80] Chaiworapongsa T (2008). Amniotic fluid heat shock protein 70 concentration in histologic chorioamnionitis, term and preterm parturition. J. Matern. Fetal Neonatal Med..

[81] Gotsch F (2008). Evidence of the involvement of caspase-1 under physiologic and pathologic cellular stress during human pregnancy: A link between the inflammasome and parturition. J. Matern. Fetal Neonatal Med..

[82] Gomez-Lopez N (2017). A role for the inflammasome in spontaneous preterm labor with acute histologic chorioamnionitis. Reprod. Sci..

[83] Gomez-Lopez N (2018). Inflammasome activation during spontaneous preterm labor with intra-amniotic infection or sterile intra-amniotic inflammation. Am. J. Reprod. Immunol..

[84] Gomez-Lopez N (2019). Inflammasomes: Their role in normal and complicated pregnancies. J. Immunol..

[85] Gomez-Lopez N (2019). Gasdermin D: Evidence of pyroptosis in spontaneous preterm labor with sterile intra-amniotic inflammation or intra-amniotic infection. Am. J. Reprod. Immunol..

[86] Motomura K (2020). The alarmin interleukin-1α causes preterm birth through the NLRP3 inflammasome. Mol. Hum. Reprod..

[87] Labbé K, McIntire CR, Doiron K, Leblanc PM, Saleh M (2011). Cellular inhibitors of apoptosis proteins cIAP1 and cIAP2 are required for efficient caspase-1 activation by the inflammasome. Immunity.

[88] Shenoy AR (2012). GBP5 promotes NLRP3 inflammasome assembly and immunity in mammals. Science.

[89] Tarca AL (2020). Crowdsourcing assessment of maternal blood multi-omics for predicting gestational age and preterm birth. bioRxiv.

[90] Jehan F (2020). Multiomics characterization of preterm birth in low- and middle-income countries. JAMA Netw. Open.

[91] Hamill N (2008). Exodus-1 (CCL20): Evidence for the participation of this chemokine in spontaneous labor at term, preterm labor, and intrauterine infection. J. Perinat. Med..

[92] Arntzen KJ, Kjøllesdal AM, Halgunset J, Vatten L, Austgulen R (1998). TNF, IL-1, IL-6, IL-8 and soluble TNF receptors in relation to chorioamnionitis and premature labor. J. Perinat. Med..

[93] Romero R (1994). Macrophage inflammatory protein-1 alpha in term and preterm parturition: Effect of microbial invasion of the amniotic cavity. Am. J. Reprod. Immunol..

[94] Bhatti G (2020). Compartmentalized profiling of amniotic fluid cytokines in women with preterm labor. PLoS One.

[95] Sadowsky DW, Adams KM, Gravett MG, Witkin SS, Novy MJ (2006). Preterm labor is induced by intraamniotic infusions of interleukin-1beta and tumor necrosis factor-alpha but not by interleukin-6 or interleukin-8 in a nonhuman primate model. Am. J. Obstet. Gynecol..

[96] Brumell JH, Craig KL, Ferguson D, Tyers M, Grinstein S (1997). Phosphorylation and subcellular redistribution of pleckstrin in human neutrophils. J. Immunol..

[97] Ding Y (2007). Phosphorylation of pleckstrin increases proinflammatory cytokine secretion by mononuclear phagocytes in diabetes mellitus. J. Immunol..

[98] Presicce P (2018). IL-1 signaling mediates intrauterine inflammation and chorio-decidua neutrophil recruitment and activation. JCI Insight.

[99] Hashimoto M (2000). Isolation and localization of type IIb Na/Pi cotransporter in the developing rat lung. Am. J. Pathol..

[100] Mittal P (2009). Fetal membranes as an interface between inflammation and metabolism: Increased aquaporin 9 expression in the presence of spontaneous labor at term and chorioamnionitis. J. Matern. Fetal Neonatal Med..

[101] Chaemsaithong P (2015). A point of care test for the determination of amniotic fluid interleukin-6 and the chemokine CXCL-10/IP-10. J. Matern. Fetal Neonatal Med..

[102] Chaemsaithong P (2016). A point of care test for interleukin-6 in amniotic fluid in preterm prelabor rupture of membranes: A step toward the early treatment of acute intra-amniotic inflammation/infection. J. Matern. Fetal Neonatal Med..

[103] Chaemsaithong P (2016). A rapid interleukin-6 bedside test for the identification of intra-amniotic inflammation in preterm labor with intact membranes. J. Matern. Fetal Neonatal Med..

[104] Honest H (2009). Screening to prevent spontaneous preterm birth: Systematic reviews of accuracy and effectiveness literature with economic modelling. Health Technol. Assess..

[105] Lee SK (2003). The benefit of preterm birth at tertiary care centers is related to gestational age. Am. J. Obstet. Gynecol..

[106] Committee on Practice Bulletins-Obstetrics, A. e. C. o. O. a. G (2004). Dystocia and augmentation of labor. Int. J. Gynaecol. Obstet..

[107] Welch BL (1947). The generalization of ‘student's' problem when several different population variances are involved. Biometrika.

[108] Fisher RAS (1934). Statistical Methods for Research Workers.

[109] Irizarry RA (2003). Exploration, normalization, and summaries of high density oligonucleotide array probe level data. Biostatistics.

[110] Carvalho BS, Irizarry RA (2010). A framework for oligonucleotide microarray preprocessing. Bioinformatics.

[111] Dai M (2005). Evolving gene/transcript definitions significantly alter the interpretation of GeneChip data. Nucleic Acids Res..

[112] Sandberg R, Larsson O (2007). Improved precision and accuracy for microarrays using updated probe set definitions. BMC Bioinform..

[113] Ritchie ME (2015). limma powers differential expression analyses for RNA-sequencing and microarray studies. Nucleic Acids Res..

[114] Falcon S, Gentleman R (2007). Using GOstats to test gene lists for GO term association. Bioinformatics.

[115] Ashburner M (2000). Gene ontology: Tool for the unification of biology. The Gene Ontology Consortium. Nat. Genet..

[116] Breiman L (2001). Random forests. Mach. Learn..

[117] Chen X, Ishwaran H (2012). Random forests for genomic data analysis. Genomics.

[118] Touw WG (2013). Data mining in the life sciences with random forest: A walk in the park or lost in the jungle?. Brief. Bioinform..

[119] Tarca AL, Carey VJ, Chen XW, Romero R, Drăghici S (2007). Machine learning and its applications to biology. PLoS Comput. Biol..

[120] Caruana, R., Karampatziakis, N. & Yessenalina, A. In *Proceedings of the 25th International Conference on Machine Learning* 96–103 (Association for Computing Machinery, Helsinki, Finland, 2008).

[121] Liaw A, Wiener M (2002). Classification and regression by random forest. R News.

[122] Gangeh MJ, Zarkoob H, Ghodsi A (2017). Fast and scalable feature selection for gene expression data using Hilbert–Schmidt independence criterion. IEEE ACM Trans. Comput. Biol. Bioinf..

[123] Doncheva NT, Morris JH, Gorodkin J, Jensen LJ (2019). Cytoscape StringApp: Network analysis and visualization of proteomics data. J. Proteome Res..

[124] Otasek D, Morris JH, Boucas J, Pico AR, Demchak B (2019). Cytoscape automation: Empowering workflow-based network analysis. Genome Biol..

